# Amino-functionalized Fe/Co bimetallic MOFs for accelerated Fe (III)/Fe (II) cycling and efficient degradation of sulfamethoxazole in Fenton-like system

**DOI:** 10.3389/fchem.2025.1579108

**Published:** 2025-03-25

**Authors:** Xianbing Zhang, Yuheng Liu, Jiajia Yuan

**Affiliations:** ^1^ National Engineering Research Center for Inland Waterway Regulation, Chongqing Key Laboratory of Ecological Waterway, Chongqing Jiaotong University, Chongqing, China; ^2^ School of Materials and Environmental Engineering, Shenzhen Polytechnic University, Shenzhen, China

**Keywords:** Fenton-like reaction, amino group introduction, dual active site, reaction mechanism, sulfamethoxazole

## Abstract

Metal-organic frameworks (MOFs) are recognized as important Fenton-like materials for environmental remediation. However, their applications are often hindered by slow cycling between Fe (III) and Fe (II). This study aimed to address the slow Fe (III)/Fe (II) cycling limitation of Fe-MOFs through dual modification strategy: bimetallic modification and amino functionalization. A series of NH_2_-MOF(Fe, Co) catalysts with varying Fe/Co ratios were synthesized via a hydrothermal method and evaluated for sulfamethoxazole (SMX) degradation. The optimized NH_2_-MOF(Fe, Co) catalyst (Fe/Co ratio = 7:3) exhibited substantially enhanced catalytic performance, with SMX removal rate and rate constant in the H_2_O_2_ system being 3.2 and 43.5 times higher than those of the Fe-MOF/H_2_O_2_ system, respectively. The catalyst demonstrated robust performance across a wide pH range (3.05–7.00), addressing a common limitation of Fenton-like systems. Physicochemical characterization revealed that the enhanced performance was attributed to two key factors: the synergistic effect between Co and Fe in the bimetallic active center, and improved electron transfer to the central metal due to -NH_2_ functionalization. These modifications effectively addressed the Fe (III)/Fe (II) redox cycling limitation. The proposed reaction mechanism provides insights into SMX degradation pathways in the NH_2_-MOF(Fe, Co)/H_2_O_2_ system. This study presents an efficient and stable MOF-based Fenton-like catalyst with potential applications in wastewater treatment and environmental remediation.

## 1 Introduction

Sulfamethoxazole (SMX) stands out as one of the most extensively used antibiotics in modern medicine, with its key information summarized in Supplementary Table S1 (Huang and Yang, 2021; Wang et al., 2024). Widely prescribed in hospital settings for the treatment a various diseases range of ailments, SMX has become a cornerstone of contemporary medical practice. However, its widespread use has led to significant environmental concerns. Conventional water and wastewater treatment processes often prove inadequate in completely remove it, resulting in its frequent detection in wastewater effluents (Pei et al., 2021; Da Silva Rodrigues et al., 2020). Consequently, SMX residues infiltrate both surface and groundwater systems, leading to a worrying accumulation of antibiotics in natural and human-habited environments, which harms non-target organisms and increases the risk of spreading drug-resistant bacteria and resistance genes (Mishra et al., 2023). Meanwhile, the high stability and antimicrobial properties of SMX further complicate its removal, particularly limiting the effectiveness of biological treatments (Suzuki et al., 2022; Kang et al., 2018).

Fenton-like technology, an advanced oxidation process, has emerged as one of the most effective chemical methods for treating organic pollutants (Wang J. F. et al., 2022; Zhang et al., 2023; Ikehata et al., 2008). Its efficacy stem from the generation of highly oxidative hydroxyl radicals (⋅OH), which rapidly and completely degrade pollutants without secondary pollution. Within this field, metal-organic frameworks (MOFs) have gained significant attention. These porous coordination polymers, formed by the self-assembly of metal ions or clusters with organic ligands, are particularly well-suited for Fenton-like technology due to their unique properties: high activity and selectivity, large specific surface area, adjustable porosity, and the ability to facility Fe^2+^/Fe^3+^ interconversion (Ma H. et al., 2021; Chen et al., 2024; Li S. et al., 2021). Recent years have seen a surge in the use of Fe-based MOFs as Fenton-like catalysts for treating recalcitrant organic pollutants. These catalysts effectively address the traditional challenges of Fenton processes, such as narrow pH range requirements and excessive iron sludge production, while achieving excellent degradation results (Sharma and Feng, 2019; Wang L. et al., 2022).

The strong interaction of H_2_O_2_ with the Fe sites in Fe-MOFs makes them react efficiently. The main challenge lies in significantly increasing the Fe (III)/Fe (II) redox rate to enhance Fe-MOFs Fenton-like properties. Various strategies have been explored, including incorporating heterogeneous materials (Du et al., 2022), construction of Fe (II)@MOFs materials (Lv et al., 2015), and utilizing light sources (Ke et al., 2019). These approaches have greatly advanced the development of MOFs materials in Fenton-like reactions and improved the efficiency of pollutant treatment. In recent years, research on bimetallic have gained popularity. Constructing a bimetallic synergistic reaction system by introducing mixed or doped metal centers (Fe/M, where M = Cu, Mn, Co, Ni) has proven effective in increasing catalytic activity. For instance, Kulandaivel synthesized Al/Fe-based MOF nanomaterials that rapidly degraded methylene blue dye within 45 min (Kulandaivel et al., 2024). Song increased the metal atom density by synthesizing Ni/Fe bimetallic cluster materials, utilizing inter-cluster for enhanced tetracycline removal (Song et al., 2023).

Functionalized of Fe-MOFs is another effective approach to enhance catalytic performance (Ma X. et al., 2021). Introducing substituents loke -OH and -NH_2_ improves pollutant adsorption by altering the catalysts’ electronic structure and increasing electron transfer (Kaur et al., 2024). For instance, NH_2_-MIL-101(Fe), NH_2_-MIL-53(Fe), and NH_2_-MIL-88B(Fe) have shown accelerated electron transfer and improved catalytic performance (Feng et al., 2023). The introduction of electrophilic amino groups the side chains can further accelerate the central metal’s redox activity and increase the catalyst’s redox potential (Chen et al., 2023). While Fe-MOFs show promise in environmental remediation, their widespread application is hindered by slow Fe (III)/Fe (II) cycling. Previous studies have explored either bimetallic synergy or functional modification separately, but few have investigated their combined effects.

Based on these considerations, we proposed a dual-modification strategy combining bimetallic synergy and amino functionalization to address the Fe (III)/Fe (II) cycling limitation in Fe-MOFs. NH_2_-MOF(Fe, Co) catalysts were synthesized via a facile hydrothermal method using 2-amino terephthalic acid as the organic linker, where the amino groups were expected to facilitate electron transfer while the Co incorporation could optimize the electronic structure of active centers. The catalytic performance was systematically evaluated using sulfamethoxazole (SMX) as a model pollutant, with particular emphasis on the optimization of Fe/Co ratio to achieve maximum synergistic effect. Comprehensive characterization techniques were employed to elucidate the structure-activity relationship, while single-factor experiments were conducted to assess the system’s adaptability to various environmental conditions. The degradation mechanism was investigated through reactive oxygen species (ROS) identification and electron paramagnetic resonance (EPR) analysis, supplemented by stability tests and interference studies to evaluate practical applicability. This study not only demonstrates an effective strategy for enhancing Fe-MOFs’ catalytic performance but also provides valuable insights into the design of efficient Fenton-like catalysts for environmental remediation, particularly in the treatment of pharmaceutical-containing wastewater under mild conditions.

## 2 Materials and methods

### 2.1 Materials and chemicals

Sulfamethoxazole (SMX), 2-Aminoterephthalic acid (2-NH_2_-BDC), tert-Butanol (TBA), p-Benzoquinone (pBQ), sodium chloride (NaCl), methanol (CH_3_OH), ethanol (C_2_H_5_OH), sodium bicarbonate (NaHCO_3_), and sodium nitrate (NaNO_3_) were procured from McLean Biochemicals Co., Ltd. N,N-Dimethylformamide (DMF), ferric chloride hexahydrate (FeCl_3_.6H_2_O), anhydrous sodium sulfate (Na_2_SO_4_), formic acid (HCOOH), and ρ-phthalic acid (H_2_BDC) were supplied by Aladdin Biochemical Technology Co., Ltd. Sinopharm Chemical Reagent Co., Ltd. Provided 30% hydrogen peroxide (H_2_O_2_), sodium hydroxide (NaOH), and nitric acid (HNO_3_). Cobalt nitrate hexahydrate (Co(NO_3_)_2_·6H_2_O) was obtained from Shanghai Acmec Biochemical Technology Co., Ltd. All chemicals and materials used in this study were analytical grade, except for methanol, which was superior purity.

### 2.2 Catalyst synthesis

**FIGURE 1:**
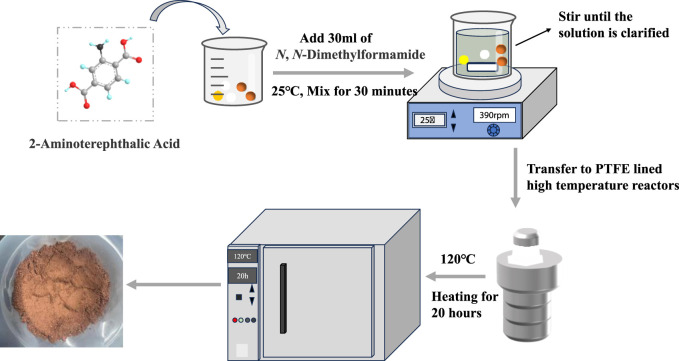
Schematic illustration of the hydrothermal synthesis process for NH_2_-MOFs(Fe, Co).

The NH_2_-MOFs(Fe, Co) catalysts were prepared using a hydrothermal method, as illustrated in Figure 1. The detailed procedures were as follows.

1) Precursor preparation, dissolve 2.50 mmol of FeCl_3_·6H_2_O and Co(NO_3_)_2_·6H_2_O in a specific molar ratio dissolved in 30 mL DMF; add 1.25 mmol of 2-NH_2_-BDC to the solution; stir the mixture magnetically at 480 rpm for 35 min at 25°C until clear.
2) Hydrothermal reaction, transfer the clear solution to a 100 mL PTFE-lined stainless-steel autoclave; heat at 120°C for 20 h; cool to room temperature for 20 h.
3) Catalysts recovery and purification, recover the catalysts and wash three times with DMF, ethanol, and deionized water; centrifuge after each wash at 6,500 rpm for 4.5 min.
4) Dry the catalysts under vacuum at 110°C for 12 h.

To optimize the metal ratio, the total amount of FeCl_3_·6H_2_O and Co(NO_3_)_2_·6H_2_O was fixed at 2.50 mmol, with Fe:Co molar ratios of 9:1, 8:2, 7:3 and 6:4.

For comparison, MOFs(Fe) and MOFs(Fe, Co) were synthesis using the same method, substituting H_2_BDC for 2-NH_2_-BDC (Raw material compositions for diverse catalysts are shown in Supplementary Table S2).

### 2.3 Characterization and analysis methods

#### 2.3.1 Morphology and structure

1) scanning Electron Microscopy (SEM) by Gemini 300 thermal field emission SEM; 2) X-ray Diffraction (XRD) by D8 Advance diffractometer (Bruker), Cu-Kα radiation, 5°–25° 2θ range, 0.4°/min scan rate.

#### 2.3.2 Chemical composition and surface properties

1) Fourier transform infrared (FTIR) spectra were obtained using a Nicolet 6700 FT-IR spectrometer with 4 cm^-1^ resolution and in range of 4,000–500 cm^-1^ at room temperature; 2) X-ray photoelectron spectroscopy (XPS) was analyzed by a Thermo Escalab 250XI (Thermo Fisher Scientific, USA) using monochromatic Al Kα radiation (hv = 1,486.6 eV) at 150 W power and a 650 µm beam spot; 3) Zeta potential at different pH values were determined by Malvern nanoparticle size potentiostat Zetasizer Pro (Zetasizer Nano ZS90, Malvern Panalytical, UK).

#### 2.3.3 Electrochemical properties

Impedance spectroscopy (EIS) was measured by an electrochemical analyzer (CHI760, Chenhua, China), 0.01 Hz to 100 kHz, 0.1 M Na_2_SO_4_ electrolyte, 12 mV with open-circuit voltage.

#### 2.3.4 Catalytic performance and reaction mechanism

1) reactive oxidants (ROS) were analyzed by a Bruker A300-10/12 electron paramagnetic resonance technique (EPR, Germany). The radical trapping agent was 5,5-dimethyl-1-pyrroline-N-oxide (DMPO); 2). The concentration of SMX was determined by HPLC with a Waters, Arc-HPLC-2998 system. The analysis employed a SunFire@C18 column (4.6 × 250 mm, 5 μm) and a mobile phase consisting of methanol and 0.1% formic acid in a 70:30 volume ratio. The flow rate was set at 0.5 mL·min^-1^, with detection performed using a UV lamp of 269 nm and a temperature of 25°C. Intermediates were identified using liquid chromatography-mass spectrometry (LC-MS, AB Sciex Triple TOF^®^4,600). The pH of solutions was adjusted by 1 mM HNO_3_ and 0.1 mM NaOH. Unless otherwise specified, the Fe/Co ratios in both NH_2_-MOFs(Fe, Co) and MOFs(Fe, Co) were maintained at 7:3 throughout the experiments.

#### 2.3.5 Stability and metal leaching

Fe and Co ion leaching after the initial cycling experiment was analyzed using AVIO 220 (PerkinElmer) inductively coupled plasma optical emission spectroscopy (ICP-OES).

### 2.4 Adsorption-catalyzed degradation experiments

The adsorption-catalyzed degradation experiments of SMX by NH_2_-MOFs(Fe, Co) was investigated at 25°C. The experiment began with 20 mL of the SMX (50 mg/L) diluted with 80 mL purified water in a 100 mL conical flask, achieving a final concentration of 10 mg/L. After 1 min of magnetic stirring at 380 rpm, a 2 mL sample was taken to determine the initial SMX concentration. The adsorption phase commenced upon adding certain concentrations of catalyst. After 30 min of adsorption of equilibrium, another 2 mL sample was collected. The catalyzed degradation was initialized by introducing H_2_O_2_. Subsequent samples were taken at 15, 30, 45, 60, 75, 90, 120, and 150 min. All samples were mixed with 200 μL of 0.5 mol/L sodium thiosulfate to quench the reaction, filtered through a 0.22 μm membrane to remove solid particles, and prepared for chromatographic analysis.

### 2.5 Cycling experiments and catalyst recovery

The catalyst’s reusability was assessed through multiple cycling experiments. After each use, the catalyst was recovered by filtration, washed with ultrapure water, and dried overnight at 60°C under vacuum conditions. The recovered catalyst was then reused in subsequent adsorption-catalyzed degradation experiments. This process was repeated several times to evaluate the catalyst’s longevity. To assess the structural and chemical changes induced by repeated use, the catalyst was analyzed before and after the reaction cycles using Fourier-transform infrared spectroscopy (FTIR), X-ray photoelectron spectroscopy (XPS), and X-ray diffraction (XRD) techniques.

## 3 Results and discussion

### 3.1 Characteristics of synthesized catalysts

**FIGURE 2:**
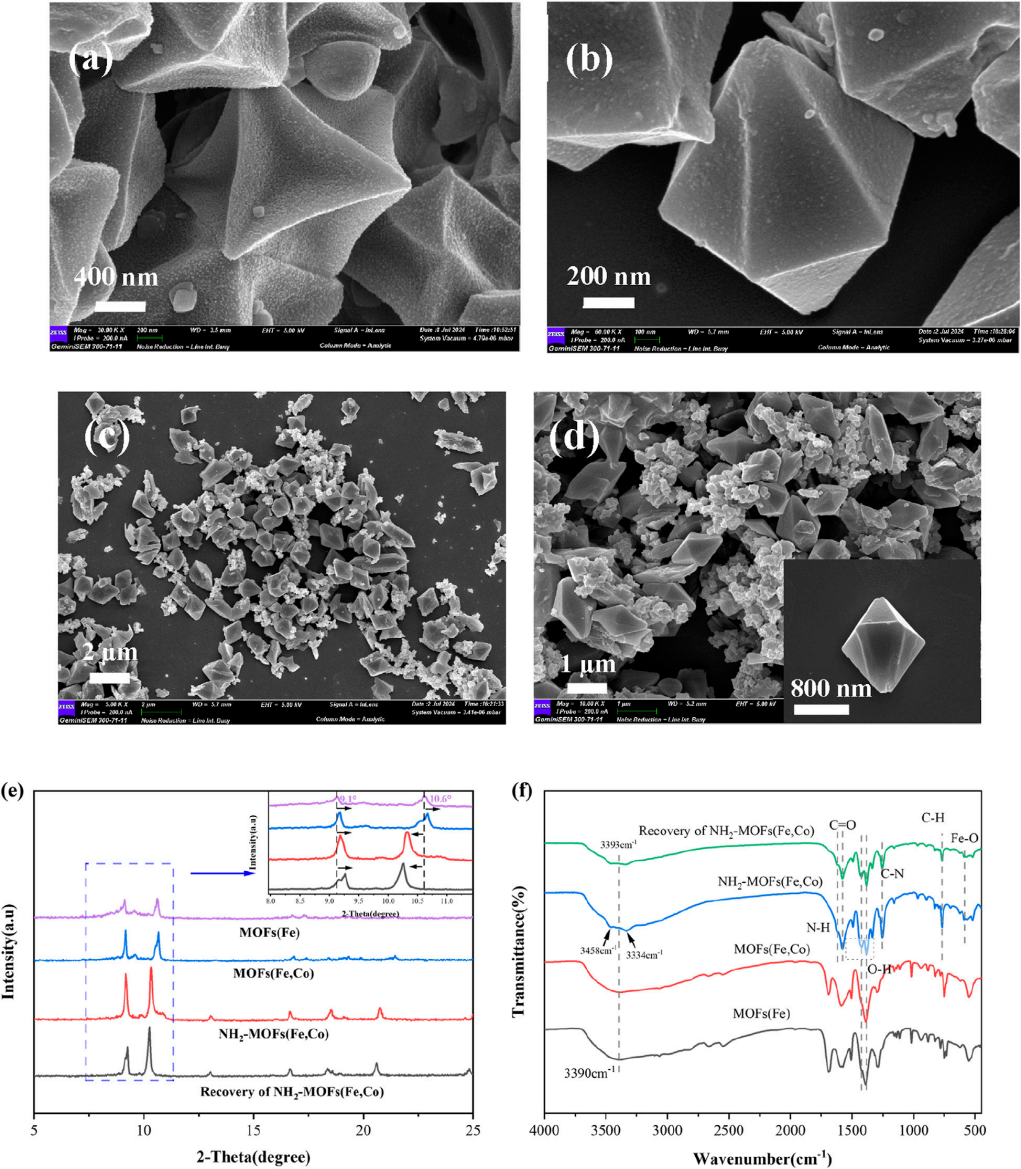
Structural and compositional analysis of MOF catalysts: **(a)** MOFs(Fe), **(b)** MOFs(Fe, Co) and **(c, d)** NH_2_-MOFs(Fe, Co). **(e, f)** XRD and FTIR patterns of MOFs(Fe), MOFs(Fe, Co) and NH_2_-MOFs(Fe, Co).

Scanning Electron Microscopy (SEM) analyses revealed the morphological characteristics of the synthesized catalysts. MOFs(Fe) displayed a three-dimensional octahedral structure with diameter of approximately 1 *μ*m (Figure 2a). The incorporation of Co atoms maintained overall morphology, the surface is concave inwards, and generally resulted in decreased particle size (Figure 2b). Amino-functionalized MOFs(Fe, Co) primarily preserved the three-dimensional octahedral structure, with a minor fraction exhibiting hexagonal micro-spindle morphology. This variation in geometric configuration suggested that ligand functionalization influenced the catalysts morphology (Figures 2c, d).

X-ray Diffraction (XRD) analysis provided insights into the crystalline structure of the synthesized catalysts (Figure 2e). MOFs(Fe) exhibited high crystallinity with distinct characteristic diffraction peaks at 2θ = 8.96°, 9.0°, 9.5°, 10.6°, and 16.5°, confirming successful synthesis of MOFs(Fe) and aligning with previous literature reports (Wu et al., 2020; Guo et al., 2019; Li et al., 2019; Gecgel et al., 2020). MOFs(Fe, Co) displayed a similar diffraction pattern to MOFs(Fe), indicating preservation of the overall structure during Co incorporation. However, slight peak shifts and intensity variations suggested successful Co doping into the MOFs(Fe) lattice.

NH_2_-MOFs(Fe, Co) exhibited distinct characteristic peaks at 2θ = 9.2°, 10.3°, 13°, 16.69°, and 18.5°, with significantly enhanced intensities in the 8°–10° and 16°–19° ranges due to -NH_2_ introduction. Compared to MOFs (Fe), peaks between 9 and 10°shifted right, while the 10.6°peak moved left, likely due to the incorporation of -NH_2_ groups and Co atoms. SEM electron micrographs corroborated the crystalline morphology of NH_2_-MOFs(Fe, Co), which showed similar alterations. Notably, NH_2_-MOFs(Fe, Co) demonstrated higher crystallinity than the other two materials (Figure 2e). Post-reaction XRD analysis revealed unchanged structures, confirming high catalyst stability and absence of significant structural disruption in NH_2_-MOFs(Fe, Co) materials.

Spectroscopic and elemental analyses of the synthesized MOF catalysts provided comprehensive characterization of their structural, compositional, and electronic properties. As shown in Figure 2f, FTIR spectra exhibited similar vibrational patterns across all samples. The absence distinct Co-O characteristic peaks after Co incorporation might be attributed to its relatively low content (He et al., 2022). Three critical spectral regions revealed essential structural information.

1) Hydroxyl Group Region (3,000–3,500 cm^−1^), the prominent absorption at 3,393 cm^−1^ corresponds to O-H stretching vibration (Yu et al., 2020), crucial for organic pollutants degradation in heterogeneous catalysis. Notably, -NH_2_ functionalization significantly enhanced this intensity in NH_2_-MOFs(Fe, Co), indicating increased surface hydroxyl groups on that provide additional reactive sites for SMX and H_2_O_2_ adsorption (Li et al., 2023). Complementary peaks at 3,458 cm^−1^ (asymmetric stretching) and 3,334 cm^−1^ (symmetric stretching) further confirmed amino group incorporation (Mohammadnezhad et al., 2021).
2) Characteristic vibrations at 1,619 cm^−1^ (N-H bending) and 1,256 cm^−1^ (C-N stretching) verified successful -NH_2_ grafting. The 1,577 cm^−1^ peak (C=O stretching) combined with bands at 1,423 cm^−1^ (symmetric O-H) and 1,382 cm^−1^ (asymmetric O-H) confirmed dicarboxylic acid ligands in the MOF architecture (Wu et al., 2021).
3) Metal-Oxygen Coordination Region (<800 cm^−1^): The 768 cm^−1^ absorption originated from C-H bending in benzene rings, while the 553 cm^−1^ feature confirmed Fe-O cluster formation in the crystalline framework (Fang et al., 2023).

X-ray Photoelectron Spectroscopy (XPS) offered detailed information on elemental compositions and electronic states. The incorporation of Co caused a shift of the Fe 2p orbital valence peaks, confirming the formation of bimetallic active center (Supplementary Figure S1) (Liang et al., 2021a). Strong N 1s signals in NH_2_-MOFs(Fe, Co) verified the successful incorporation of amino groups. Shifts in binding energies of C 1s, O 1s, and Fe 2p peaks further validated the synthesis of NH_2_-MOFs(Fe, Co). Notably, the introduction of Co and -NH_2_ groups enhanced electron transfer from ligands to metal centers, as evidenced by shifts in binding energies of the C 1s, O 1s, and Fe 2p peaks to varying extents.

**FIGURE 3:**
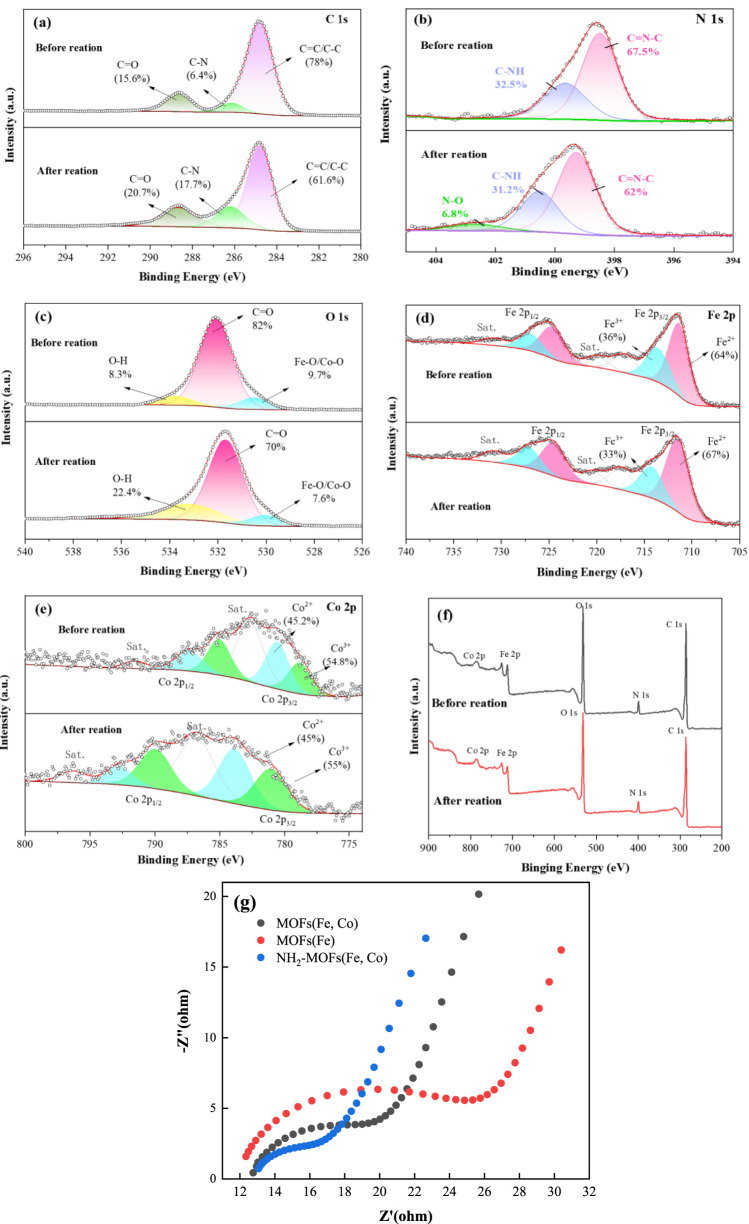
XPS and EIS Analysis of NH_2_-MOFs(Fe, Co): **(a)** C 1s **(b)** N 1s **(c)** O 1s **(d)** Fe 2p **(e)** Co. 2p **(f)** survey scan and **(g)** EIS Nyquist plots of MOFs(Fe), MOFs(Fe, Co) and NH_2_-MOFs(Fe, Co).

High-resolution XPS spectra provided detailed insights into the electronic structure and chemical composition of NH_2_-MOFs(Fe, Co) catalysts before and after reaction. The C 1s spectrum revealed three distinct peaks corresponding to C=C/C-C, C-N, and C-O bonds (Figure 3a) (Zhou and Wang, 2023). Post-reaction changes in peak intensities indicated active involvement of carbon species in the catalytic process. After the reaction, the relative intensities of these peaks changed by 16.4%, 11.3%, and 5.1%, respectively. The N 1s spectrum (Figure 3b) demonstrated significant chemical transformation in NH_2_-MOF (Fe, Co.) during the Fenton reaction. Prior to the reaction, two distinct nitrogen species were identified: a C-NH configuration at 399.08 eV (32.5%) and a C=N-C at 400.14 eV (67.5%). Post-reaction analysis revealed a marked reduction in the contents of both components (C-NH: 32.5% → 28.1%; C=N-C: 67.5% → 65.1%), indicative of structural degradation or chemical modification. Notably, a new N-O species emerged at 402.67 eV (6.8%), confirming the oxidation of amino group and the formation of nitrogen-oxygen compounds. This evolution directly implies amino group participation in the Fenton reaction mechanism through electron transfer processes (Zeng et al., 2017). The O 1s spectrum (Figure 3c) displayed peaks corresponding to metal oxides, carboxylate C-O bonds, and O-H bonds. A reduction in metal-oxygen bond content after reaction indicated electron transfer from O^2-^ to Fe (II) during oxidation.

Analysis of the Fe 2p orbital revealed peaks corresponding to Fe^2+^ and Fe^3+^ in both Fe 2p3/2 and Fe 2p1/2 regions. The stable distribution of Fe species before and after reaction demonstrated the redox stability of iron in the catalyst. Notably, the introduction of -NH_2_ groups caused a red-shift in Fe-O/Co-O peak binding energies (Supplementary Figure S1) and a slight shift of Fe 2p orbital valence peaks towards lower binding energies (Liang et al., 2021b). These shifts suggest that -NH_2_ functionalization alters the material’s electronic structure, enhancing electron transfer from the ligand to the central metal (Li et al., 2017).

The Co 2p spectrum (Figure 3e) showed two characteristic peaks attributed to Co^3+^ (780.7 eV and 789.3 eV) and Co^2+^ (783.1 eV and 792.6 eV), with a better cycling state of Co species before and after the reaction.

Additionally, EIS results demonstrated that the incorporation of dual reaction centers and -NH_2_ enhanced the material’s interfacial electron transfer capacity (Figure 3g).

These results collectively provide valuable insights into the electronic properties and reaction mechanisms of NH_2_-MOFs(Fe, Co) catalysts, highlighting the role of amino functionalization in modifying the catalyst’s electronic structure and potentially enhancing its catalytic performance.

### 3.2 Adsorption-catalyzed degradation properties of catalysts

**FIGURE 4:**
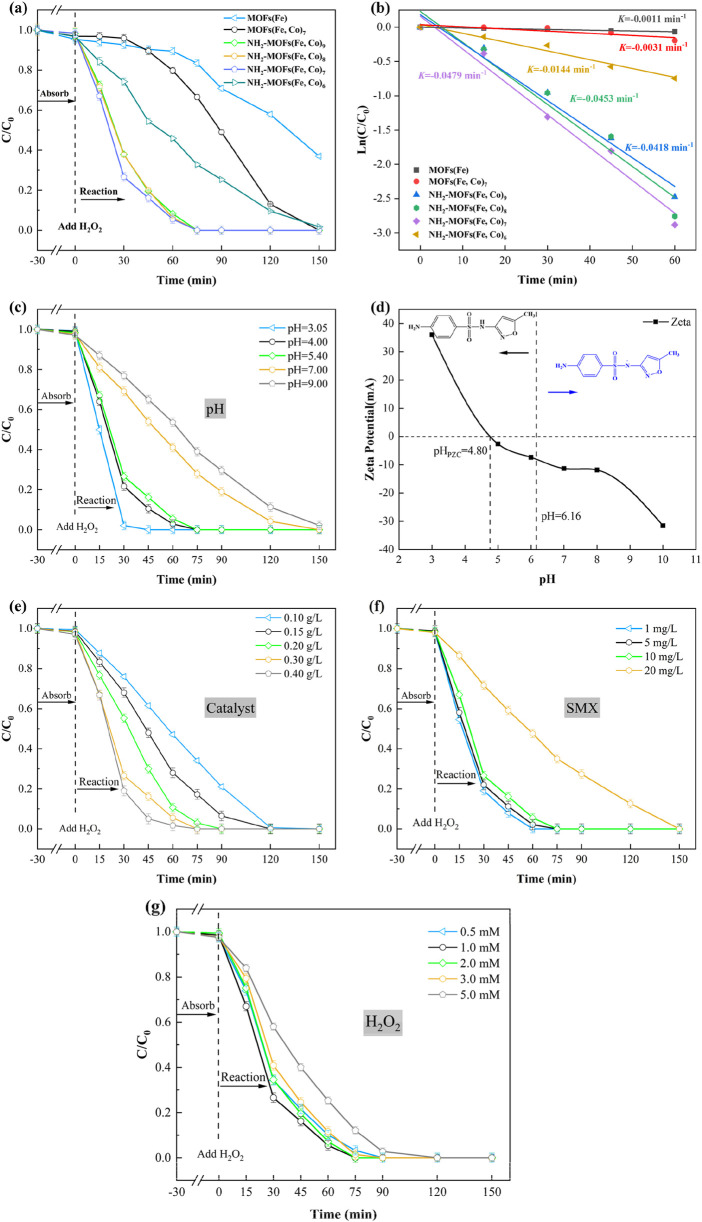
Degradation curves of SMX in different reaction systems **(a)** and the corresponding kinetic curves **(b)**. The effect of **(c)** pH value for SMX degradation in the NH_2_-MOFs(Fe, Co)/H_2_O_2_ system. **(d)** The Zeta potential of NH_2_-MOFs(Fe, Co) under different pH value. The effect of **(e)** catalyst dosage, **(f)** concentration of SMX, and **(g)** concentration of H_2_O_2_ for SMX degradation in the NH_2_-MOFs(Fe, Co)/H_2_O_2_ system (Reaction conditions: natural pH value (ca. 5.4 ± 0.1), initial SMX concentration: 10 mg/L, H_2_O_2_ concentration: 1mM, and catalyst dosage: 0.3 g/L. where X represents the ratio of iron.).

A series of experiments were conducted to evaluate the performance of bimetallic NH_2_-MOFs(Fe, Co) as Fenton-like catalysts for SMX degradation (Test method for the concentration of SMX is given in Supplementary Text S1). The study revealed that adsorption played a minimal role, with less than 5% of SMX being removed across all metal ratios (Figure 4a). Similarly, H_2_O_2_ alone proved ineffective in degrading SMX, due to its relatively low oxidation potential (1.776 V) (Li et al., 2024; Ramos et al., 2021).

Comparative analysis of MOFs(Fe) and MOFs(Fe, Co) under identical conditions demonstrated the significant impact of Co incorporation on catalytic performance. While MOFs(Fe) achieved 63.1% removal of SMX after 150 min, MOFs(Fe, Co) removed 87% of SMX after 120 min and completely removal within 150 min. This enhanced performance is attributed to the synergistic interaction between Fe and Co within the catalyst structure. The introduction of Co into MOFs(Fe) optimizes the electronic structure, lower the energy barrier for H_2_O_2_ activation, and promotes increased hydroxyl radicals (⋅OH). These factors contribute to more efficient SMX degradation (Liang et al., 2021a). Furthermore, Co can undergo redox reaction with H_2_O_2_ like Fe, further enhancing the catalytic process (Equation 1).

$$\text{Co}\left(\text{II}\right)+H_{2}O_{2}\to \text{Co}\left(\text{III}\right)+\cdot \text{OH}+{\text{OH}}^{‐}$$ (1)

The introduction of -NH_2_ greatly to MOFs(Fe, Co) catalyst resulted in a remarkable improvement in their performance for SMX degradation. Comparative studies revealed that while MOFs(Fe, Co) removed only 33.5% of SMX in 75 min, the amino-functionalized NH_2_-MOFs(Fe, Co) achieved completely removal in same timeframe. Furthermore, in the presence of H_2_O_2_, NH_2_-MOFs(Fe, Co)x were all able to completely degrade SMX within 150 min. This significant enhancement in catalytic activity can be attributed to several factors. Firstly, the amino group facilitates accelerated electron transfer within the catalyst structure, promoting faster valence transitions of the central metal atoms. Secondly, it increases the electron density around the metal centers, enabling continuous and efficient production of hydroxyl radicals (⋅OH) (Chen et al., 2023). Furthermore, the incorporation of Co and -NH_2_ may facilitate the formation of Lewis acid sites, thereby providing more active sites for H_2_O_2_ activation (Liang et al., 2021b; Huang et al., 2022).

The Fe: Co ratio in metal-organic framework (MOF) catalysts significantly influences their performance in SMX degradation. Adjusting the ratio from 9:1 to 7:3 increased the SMX degradation rate from 91.8% to 94.5%, demonstrating a synergistic effect between Fe and Co However, further increasing the Co content to a 6:4 ratio led to a substantial decrease in catalytic performance, with only 54.2% SMX degradation at 60 min. This decline in performance with excessive Co doping can be attributed to two main factors: 1) excessive Co^2+^ occupying the active sites belonging to Fe^2+^, inhibiting the catalyst’s activity (Li et al., 2024); 2) high cobalt content could potentially compromise the material’s structural stability.

The catalytic efficiency evaluation revealed a clear performance hierarchy among the tested MOF catalysts in Fenton-like reactions: MOFs(Fe) < MOFs(Fe, Co) < NH_2_-MOFs(Fe, Co). This progression underscores the synergistic enhancement from bimetallic composition and amino functionalization. The NH_2_-MOFs (Fe, Co)/H_2_O_2_ system showed greater superiority over the existing catalysts (Supplementary Table S3). First, it achieved complete degradation of 10 mg/L SMX within 75 min under natural pH (5.4), surpassing MIL-53(Fe) (96% removal of 200 μg/L SMX at pH 4 with UV assistance) (Ortega-Moreno et al., 2022), CUCs-MIL-88 B-Fe/Ti_3_C_2_ (96% removal of 30 mg/L SMX requiring visible light) (Ahmad et al., 2020), and Fe@MesoC (100% removal of 20 mg/L SMX at pH 4) (Tang and Wang, 2018). Second, operational efficiency was optimized through reduced reagent demands—0.3 g/L catalyst dosage and 1 mM H_2_O_2_ concentration, significantly lower than literature values (200–500 mg/L catalyst, 2–10 mM H_2_O_2_) (Tang and Wang, 2020) while eliminating energy-intensive UV/visible light requirements. Third, the system demonstrated exceptional environmental adaptability by maintaining efficacy without pH adjustment, contrasting sharply with conventional Fenton systems constrained to acidic conditions (pH 2.8–3.5). These advancements position NH_2_-MOFs(Fe, Co) as a practical solution for wastewater remediation. Subsequent research prioritized two key aspects: rigorous stability evaluation through multi-cycle testing and mechanistic investigation via radical trapping/LC-MS analysis, establishing a foundation for industrial implementation.

The degradation kinetics of SMX were evaluated using pseudo-primary rate constants, calculated according to Equation 2. Figure 4b presents a comparative analysis of the kinetics across different reaction systems, offering insights into their relative efficiencies. Among the systems studied, the NH_2_-MOFs(Fe, Co)/H_2_O_2_ system exhibited the highest rate constant (*k*-value) of 0.0479 min^-1^, significantly outperforming the other ones. The observed enhancement can be attributed to several factors. Firstly, the amino group accelerates the cycling of the central metal, facilitating more efficient catalytic turnover. Secondly, it alters the electronic structure of the catalyst, potentially creating more favorable conditions for the degradation reaction. Lastly, the -NH_2_ group promotes more effective activation of H_2_O_2_, a crucial component in the degradation process

$$\ln\left(C_{t}/C_{0}\right)=-\text{kt}$$ (2)

where *C*_t_ (mg/L) is the concentration of SMX at a certain reaction time *t* (min), and *C*_0_ (mg/L) refers to the concentration of SMX after the adsorption-desorption equilibrium is reached and before oxidative degradation begins.

### 3.3 Effect of key parameters on SMX degradation

The effectiveness of Fenton-like systems in degrading organic pollutants is heavily influenced by several key parameters, including initial pH, catalyst amount, initial pollutant concentration, and H_2_O_2_ content (Dapaah et al., 2022). To optimize these factors for SMX degradation, we conducted a series of controlled experiments using simulated wastewater, with results presented in Figures 4c–f.

As shown in Figure 4c, the initial pH plays a crucial role in the degradation process, though it has minimal impact on SMX adsorption by NH_2_-MOFs(Fe, Co). The system demonstrated optimal performance in the pH range of 3.0–5.4, achieving complete SMX removal within 75 min. Efficiency decreased at a higher pH level, primarily due to the formation and decomposition of hydroxides inhibiting iron species reactivity, as well as reduced H_2_O_2_ availability and its self-decomposition of H_2_O_2_ (Tang and Wang, 2019). Zeta potential analysis revealed a point of zero charge (pH_PZC_) 4.8 for NH_2_-MOFs(Fe, Co), indicating a negatively charged catalyst surface above this pH and a positively charged surface below it (Figure 4d). The ionization state of SMX also varies with pH, existing in an amphipathic form (SMX±) between pH 1.97 and 6.16, and as an anionic species (SMX-) above pH 6.16 (Brillas et al., 2009). Notably, even at a pH of 9, the system achieved nearly 90% SMX removal after 120 min reaction, significantly outperforming the typical optimal pH range (2.8–3.5) for conventional Fenton reactions (Milh et al., 2020). The reduced SMX degradation efficiency under alkaline conditions (pH > 8) likely arises from electrostatic repulsion between the negatively charged surface and deprotonated SMX molecules (p*K*a = 5.7), as a evidenced by the surface charge reversal observed in zeta potential measurements.

Based on these results, we selected the unadjusted initial pH of 5.4 for subsequent optimization studies, as it demonstrated excellent performance with complete SMX removal within 75 min. This pH level strikes a balance between optimal catalytic activity and practical applicability, potentially reducing the need for pH adjustment in real-world applications.

Figure 4e presents the experimental results of initial catalyst concentration (0.1–0.4 g/L) on pollutant removal. Notably, all tested concentrations achieved near-complete SMX degradation within 120 min, highlighting the system’s robustness across a range of catalyst loading. The study revealed a nuanced relationship between catalyst concentration and degradation efficiency. While the removal rate at 90 min initially increased with higher catalyst concentrations, this trend showed diminishing returns at higher levels. A catalyst concentration of 0.3 g/L emerged as the optimal point, achieving approximately 95% SMX removal, which was comparable to the performance at 0.4 g/L. Interestingly, while the catalyst concentration had a minimal effect on adsorption performance, it demonstrated a positive correlation with catalytic degradation efficiency. This relationship can be attributed to the increased availability of active sites at higher concentrations, which accelerates the production of OH radicals during SMX degradation, as noted by Guo et al. (2020). Therefore, a catalyst concentration of 0.3 g/L was selected as the condition for the subsequent experiments.

The impact of SMX concentration on the catalytic degradation efficiency was investigated (Figure 4f). The results revealed an inverse relationship between SMX concentration and removal efficiency. This phenomenon can be attributed to reduced contact efficiency between the catalyst’s active sites and SMX molecules at higher concentrations, as suggested by Li et al. (2024).

Figure 4g demonstrates the effect of H_2_O_2_ concentration on SMX degradation efficiency, showing a non-linear relationship. As the H_2_O_2_ concentration increased from 0.5 mM to 1 mM, the SMX removal rate increased. However, further increase in H_2_O_2_ concentration led to a gradual decrease in removal efficiency. This trend can be explained by the dual role of H_2_O_2_ in the process. At appropriate concentrations, H_2_O_2_ enhances the production of free radicals, improving SMX degradation. Conversely, excessive H_2_O_2_ can reduce Fenton-like activity and even quench ⋅OH radical, as described by Equations 3 and 4 (Yang et al., 2021). These findings highlight the importance of optimizing H_2_O_2_ dosage to balance treatment effectiveness and cost efficiency.

Based on the above results and cost-saving, the optimal conditions for SMX degradation were determined as follows: initial SMX concentration of 10 mg/L, NH_2_-MOFs(Fe, Co) concentration of 0.3 g/L, H_2_O_2_ concentration of 1 mM and an operation pH at 5.4.

### 3.4 Stability and reusability of NH_2_-MOFs(Fe, Co)

**FIGURE 5:**
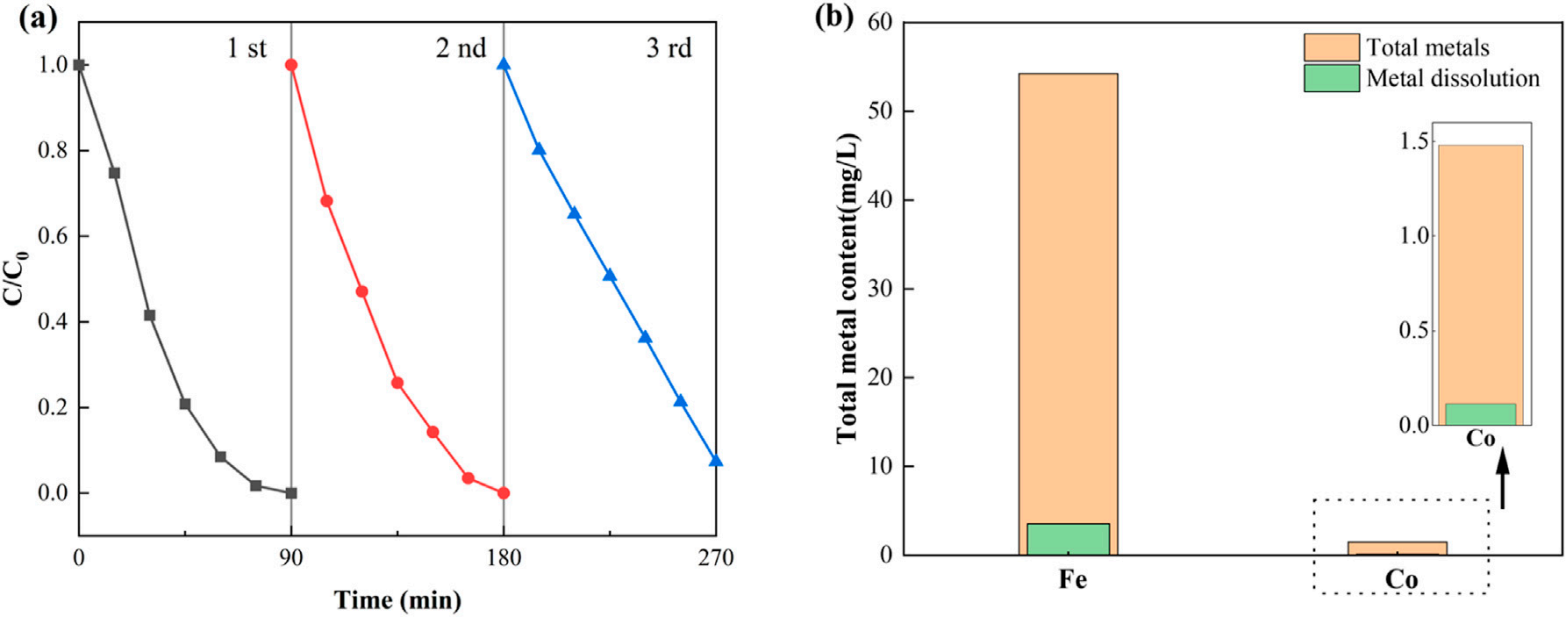
**(a)** Recyclability of NH_2_-MOFs(Fe, Co) in Fenton-like degradation of SMX, **(b)** total metal content and 90 min dissolution. Experiment conditions: natural pH (ca.5.4 ± 0.1), initial SMX concentration:10 mg/L, H_2_O_2_ concentration: 1mM, catalyst dosage: 0.3 g/L, and temperature 25°C.

The practical applicability of the NH_2_-MOFs(Fe, Co) catalyst was assessed through reusability tests and stability analyses. Consecutive cycling tests for SMX removal were conducted under consistent conditions (Initial SMX concentration: 10 mg/L, concentration of NH_2_-MOFs(Fe, Co): 0.3 g/L, H_2_O_2_ concentration: 1mM, pH = 5.4). Figure 5a illustrates the catalyst’s reusability performance. NH_2_-MOFs(Fe, Co) maintained stable catalytic efficiency though the first two cycles. A slight decrease in performance was observed in the third cycle, the catalyst activity stabilised and did not decrease during the cycle. This pattern indicates robust and sustainable catalytic activity over multiple uses.

The stability of the catalyst was further confirmed by metal leaching tests and post-reaction analyses. As shown in Figure 5b, less than 10% metal leaching occurred after 90 min of reaction, suggesting high structural integrity of NH_2_-MOFs(Fe, Co). Moreover, XRD, FTIR, and XPS analyses of the used catalyst revealed no significant changes in its internal chemical bonding or electronic structure.

**FIGURE 6:**
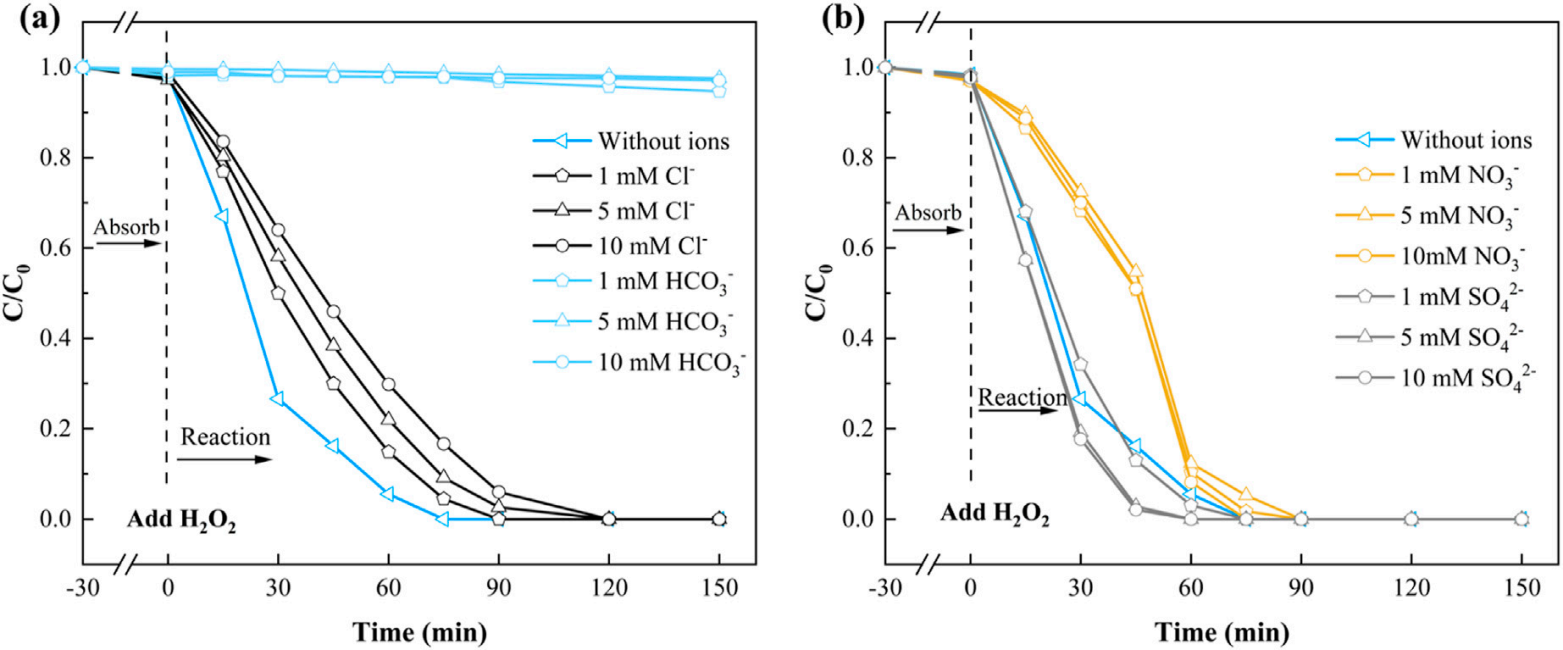
The effect of **(a)** Cl^−^,HCO_3_^−^; **(b)** NO_3_^−^, SO_4_^2-^ of NH_2_-MOFs(Fe, Co) in Fenton-like degradation of SMX. Reaction conditions: natural pH = 5.4 ± 0.1, initial SMX concentration: 10 mg/L, H_2_O_2_ concentration: 1mM, catalyst dosage: 0.3 g/L, and temperature 25°C.

The presence of inorganic anions in natural waters can significantly influence H_2_O_2_ activation and organic pollutants removal (Wang et al., 2021; Su et al., 2022). This study examined the effects of common anions (Cl^−^, HCO_3_^−^, NO_3_^−^, and SO_4_^2-^) on SMX removal in the NH_2_-MOFs(Fe, Co.)/H_2_O_2_ system. Cl^−^ exhibited a concentration-dependent inhibitory effect on SMX removal within the 1–10 mM (Figure 6a). Despite this, the catalyst maintained high activity, achieving complete SMX degradation within 120 min even at 10 mM Cl^−^. This inhibition is attributed to Cl^−^—as a radical scavenger, forming less reactive Cl·, Cl_2_·, and HClO·^-^—upon reaction with ⋅OH (Goulart et al., 2021).

HCO_3_^−^ demonstrated a more pronounced inhibitory effect (Figure 6a). At just 1 mM HCO_3_^−^, SMX removal rate dropped below 5%. This strong inhibition results from HCO_3_^−^ reacting with ⋅OH to form less effective radical species (Lin et al., 2019).

NO_3_^−^ and SO_4_^2-^ showed contrasting effects (Figure 6b). NO_3_^−^ exhibited a consistent inhibitory effect across the 1–10 mM range, though complete SMX removal was still achieved within 90 min. Conversely, SO_4_^2-^ promoted SMX degradation, with increasing effectiveness up to 5 mM concentration, further increases in SO_4_^2-^ concentration did not significantly enhance system performance.

### 3.5 Catalytic mechanisms

**FIGURE 7:**
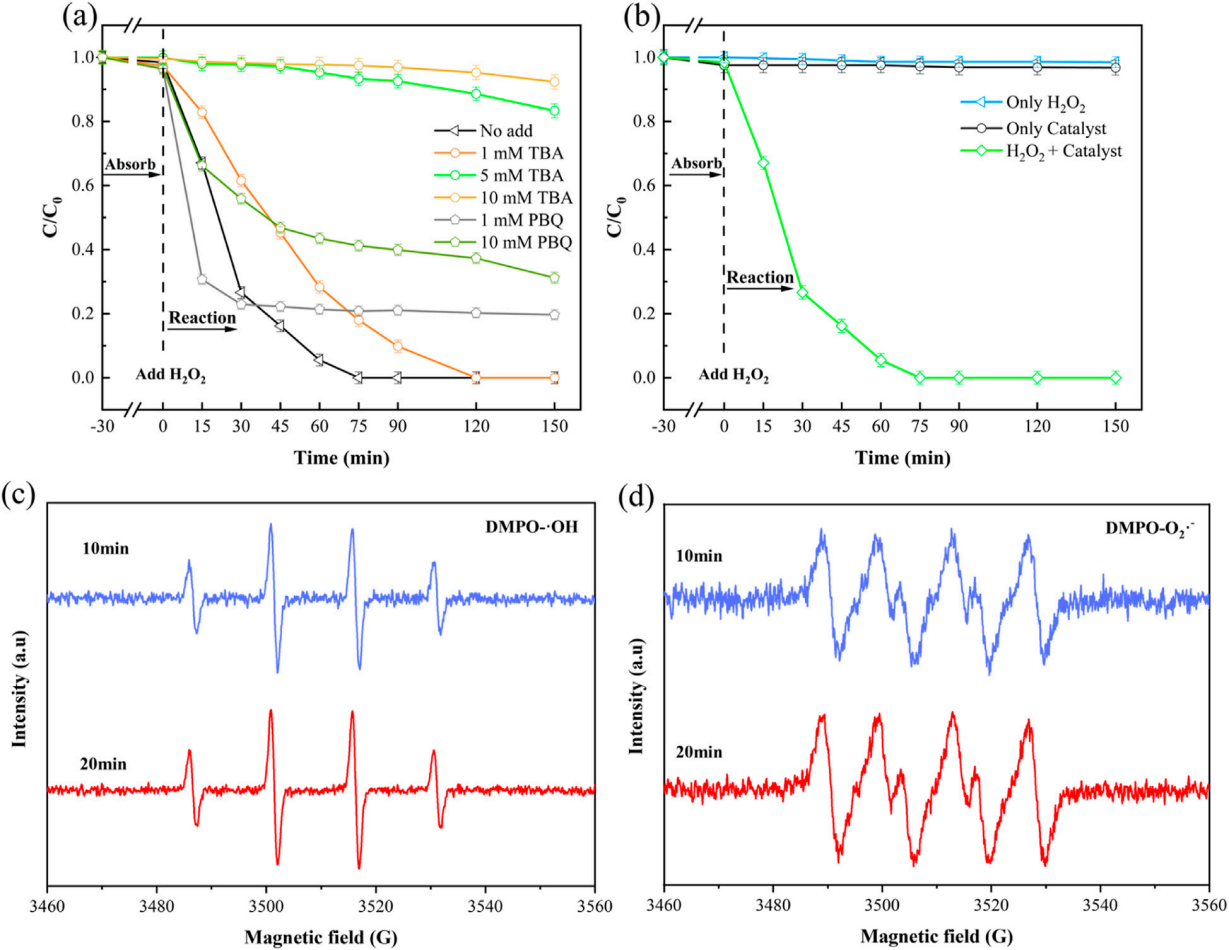
Radical scavenging experiments and EPR analysis of the NH_2_-MOFs(Fe, Co)/H_2_O_2_ system for SMX degradation. **(a)** Effects of different concentrations of trapping agents TBA and pBQ on the degradation of SMX simulated wastewater by NH_2_-MOFs(Fe, Co)/H_2_O_2_ system; **(b)** Effect of catalysts and oxidizers on SMX degradation; **(c)** DMPO-⋅OH spin-trapping EPR spectra; **(d)** DMPO-⋅O_2_^−^ spin-trapping EPR spectra. Reaction conditions: natural pH = 5.4 ± 0.1, initial SMX concentration:10 mg/L, H_2_O_2_ concentration: 1mM, catalyst dosage: 0.3 g/L, and temperature 25°C).

In Fenton-like systems utilizing MOF materials, the activation of H_2_O_2_ primarily generates two reactive oxygen species (ROS): ⋅OH and ⋅O_2_^−^. Among these, ⋅OH is generally considered the principle active species responsible for pollutant degradation (Li et al., 2023). To elucidate the roles of these ROS in our NH_2_-MOFs(Fe, Co)/H_2_O_2_ system, tert-butanol (TBA) and p-benzoquinone (pBQ) were employed as specific scavengers to quench ⋅OH and ⋅O_2_^−^ respectively. As shown in Figure 7a, the addition of 1 mM TBA reduced SMX rate, though completely degradation was still achieved within 120 min. Increasing TBA concentration to 10 mM significantly inhibited SMX degradation, with only 5% removal after 150 min. In contrast, the addition of 10 mM pBQ allowed for nearly 70% SMX degradation within 150 min. These results clearly demonstrate that ⋅OH is the primary active radical responsible for SMX elimination in this system.

The synergistic action of the catalyst and oxidant in SMX degradation was demonstrated through control experiments and Electron Paramagnetic Resonance (EPR) spectroscopy (Specific test conditions are shown in Supplementary Text S2). Figure 7b shows that neither H_2_O_2_ nor the catalyst alone significantly degraded SMX, confirming that the degradation process relies on reactive radicals generated through their combined action.

EPR analysis was employed to identify and quantify the active species produced during H_2_O_2_ activation. DMPO (5,5-dimethyl-1-pyrroline N-oxide) was used as a spin-trapping agent for both hydroxyl (⋅OH) and superoxide (⋅O_2_^−^) radicals. As shown in Figure 7c, the characteristic 1:2:2:1 peak pattern of DMPO-⋅OH adducts was observed, with strong signal intensity maintained at 10 and 20 min. This aligns with the SMX degradation trends observed in previous experiments. Additionally, DMPO-⋅O_2_^−^ signals were detected (Figure 7d), indicating the presence of superoxide radicals. Notably, the introduction of the -NH_2_ group in the MOF structure enhanced ⋅O_2_^−^ production, potentially leading to increased generation of reactive radicals. This observation is consistent with previous studies (Huang et al., 2022) and suggests that the amino functionalization of the MOF plays a crucial role in the system’s catalytic efficiency.

**FIGURE 8:**
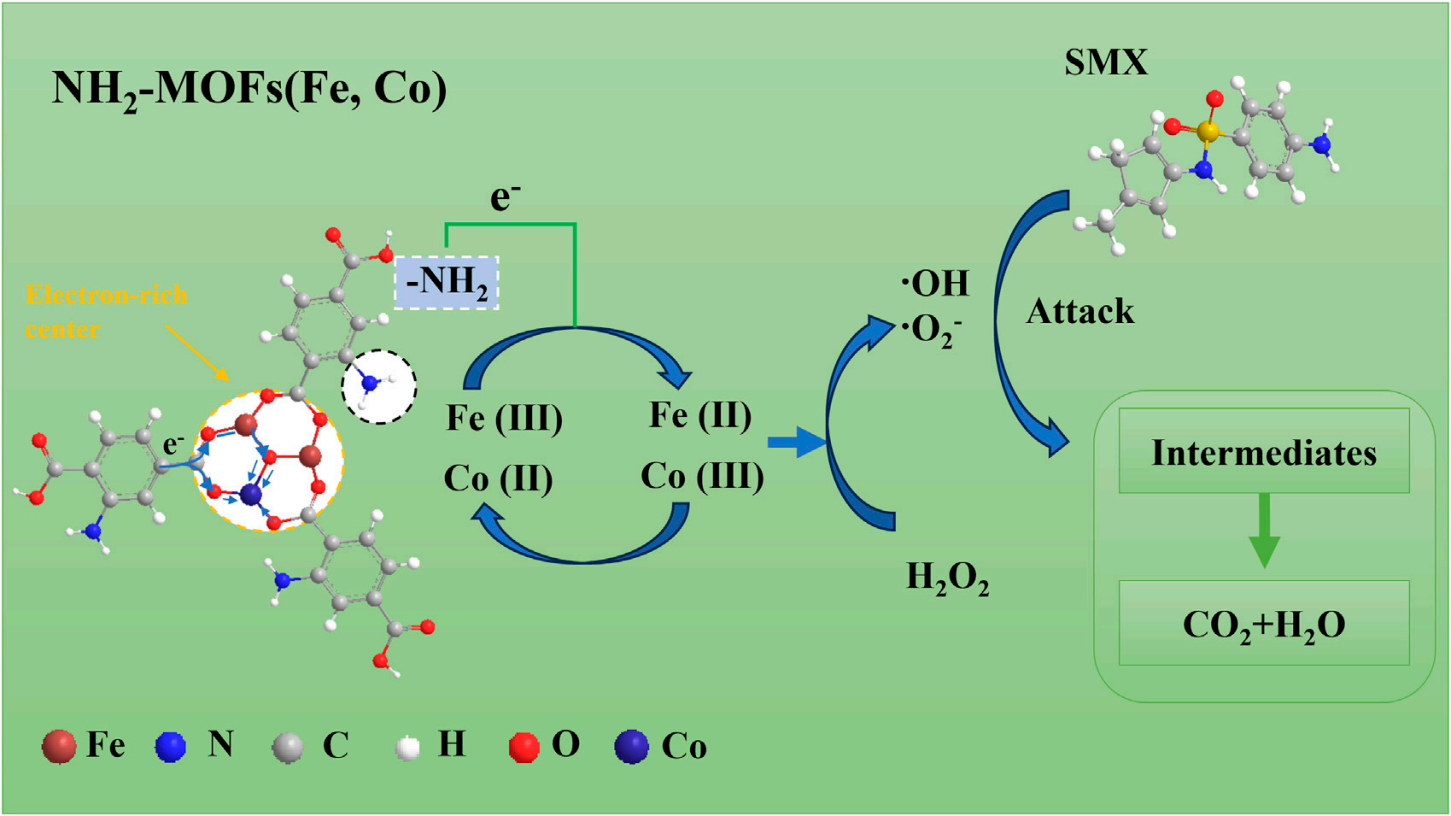
Schematic diagram of the proposed mechanisms involved for SMX degradation in NH_2_-MOFs(Fe, Co)/H_2_O_2_ system.

Based on the above results, a rational mechanism for the Fenton-like NH_2_-MOFs(Fe, Co) reaction could be proposed (As shown in Figure 8). The catalyst’s dual active metal sites, Fe and Co, undergo valence changes between Fe (III)/Co (III) to Fe (II)/Co (II) states (Equations 5, 6) (Liang et al., 2021a). This process facilitates the activation of adsorbed H_2_O_2_, generating ⋅OH and ⋅O_2_^−^ radicals through Fenton-like reactions (Equations 7, 8).

The efficiency of this process is enhanced by several factors: 1) π-bonds between SMX and NH_2_-MOFs(Fe, Co) improve the pollutant’s attachment to the catalyst surface (Cui et al., 2021), promoting reactions with surface-bound radicals (Liu et al., 2021; Xiao et al., 2023); 2) the electron-rich -NH_2_ group reduces the electron density of the intermediate metal, accelerating both the Fe (III)/Co (III) to Fe (II)/Co (II) transition and H_2_O_2_ decomposition; 3) XPS data confirm stable cycling of the central metals within NH_2_-MOFs(Fe, Co), with Fe (II) and Co (III) ratios remaining relatively constant post-reaction (Figure 3). The continuous electron extraction from NH_2_-MOFs(Fe, Co) by H_2_O_2_ produce sustains ⋅OH production. Subsequently, the combined action of ⋅OH and ⋅O_2_^−^ radicals decomposes intermediates into CO_2_ and H_2_O (Huang et al., 2023).

### 3.6 Degradation pathways of SMX

**FIGURE 9:**
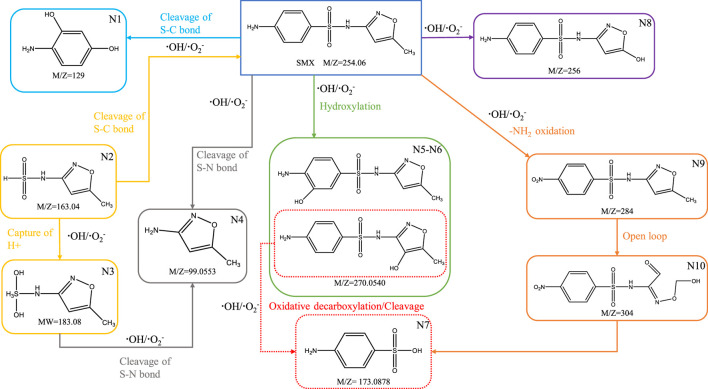
A possible pathway of SMX degradation by NH_2_-MOFs(Fe, Co)/H_2_O_2_.

To elucidate the degradation mechanism of SMX in the NH_2_-MOFs(Fe, Co)/H_2_O_2_ system, the formed intermediates were characterized using TOF (Analysis parameters are shown in Supplementary Text S3). Ten primary intermediates (N1-N10) were detected and characterized (Supplementary Table S4). The potential decomposition pathways of SMX were proposed based on the reaction properties of ⋅OH and ⋅O_2_^−^ radicals, as illustrated in Figure 9. The strong redox capacity ⋅OH/⋅O_2_^−^ primary targets the S-C and S-N bonds in SMX (Wang and Wang, 2017). S-C bond cleavage leads to the formation of N1 (m/z = 129) and N2 (m/z = 163.04), while S-N bond breakage produces N4 (m/z = 99.0553). These observations align with previous studies by Wang and Wang. (2017), Milh et al. (2020), and Liu et al. (2017) (Wang and Wang, 2017; Milh et al., 2020; Liu et al., 2017). N2 can further generate N3 (m/z = 183.08) by binding H^+^ from water and the same reaction has been reported in previous studies (Li T. et al., 2021). Hydroxylation of benzene and isoxazole rings by ⋅OH attack results in N5 and N6 (m/z = 270), influenced by the electron-donating effect of -NH_2_ and electron-withdrawing effect of -S(O_2_)-NH- groups, as noted by Ribeiro (Ribeiro et al., 2016). Further transformations include demethylation and hydroxylation leading to N8 (m/z = 256), and oxidative decarboxylation converting N6 to N7 (m/z = 173.0878), consistent with findings by Chen (Chen et al., 2023). The formation of nitro derivatives (N9, m/z = 284) occirs through ⋅OH radical attack on the -NH_2_ group, forming hydroxylamine and subsequently nitroso derivatives, as described by Milh (Milh et al., 2021). Additionally, isoxazole ring (N10, m/z = 304) opening followed by oxidation/decarboxylation or sulfonamide bond breakage leads to the formation of N7 (m/z = 173.0878), aligning with previous studies on H_2_O_2_ oxidation catalyzed by functionalized iron MOFs (Chen et al., 2023).

This study presents a significant advance in the development of Fenton-like catalysts by successfully combining bimetallic synergy with amino functionalization in MOF materials. Although this study has established the fundamental correlation between structural modifications and enhanced performance, several aspects warrant further investigation: (1) *in-situ* characterization of the electron transfer process between metal centers and amino groups; (2) molecular-level understanding of the Co-Fe synergistic mechanism; and (3) catalyst behavior in complex environmental matrices. Future research should focus on scaling up synthesis procedures, evaluating performance in real pharmaceutical wastewater treatment, and developing strategies for catalyst regeneration and recycling. Additionally, the catalytic performance and structural stability of NH_2_-MOFs(Fe, Co) exhibited strong dependence on vacuum drying temperatures during synthesis. Systematic optimization revealed elevated temperatures enhanced crystallinity and framework integrity, with 110°C identified as the optimal condition for balancing thermal stability and active site preservation.

The insights gained from this study not only contribute to the rational design of efficient MOF-based catalysts but also offer promising solutions for sustainable wastewater treatment, particularly in addressing emerging pharmaceutical pollutants under environmentally relevant conditions.

$$H_{2}O_{2}+\cdot \text{OH}\to H_{2}O+\cdot HO_{2}$$ (3)

$$\cdot HO_{2}+\cdot \text{OH}\to H_{2}O+O_{2}$$ (4)

$$\text{Fe}\left(\text{III}\right)+e^{‐}\to \text{Fe}\left(\text{II}\right)$$ (5)

$$\text{Co}\left(\text{III}\right)+e^{‐}\to \text{Co}\left(\text{II}\right)$$ (6)

$${\text{Fe}}^{3+}+H_{2}O_{2}\to {\text{Fe}}^{2+}+\cdot O_{2}^{-}+2H^{+}$$ (7)

$${\text{Co}}^{3+}+H_{2}O_{2}\to {\text{Co}}^{2+}+\cdot O_{2}^{-}+2H^{+}$$ (8)

## 4 Conclusion

In this study, we successfully developed an amino-functionalized bimetallic MOF catalyst [NH_2_-MOF(Fe, Co)] through a facile hydrothermal synthesis method for enhanced Fenton-like degradation of sulfamethoxazole (SMX) pollutants. The optimized catalyst demonstrated remarkable advantages over conventional Fe-MOFs in SMX removal, achieving 95% degradation within 120 min under neutral pH conditions (0.3 g/L catalyst, 1 mM H_2_O_2_). Notably, the catalytic efficiency and kinetic rate constant were enhanced by 3.2 and 43.5 times, respectively, compared to the Fe-MOF/H_2_O_2_ system at natural pH, addressing a critical limitation of traditional Fenton processes.

Systematic investigation of operational parameters revealed the catalyst’s robust performance across a wide pH range and excellent stability over multiple cycles. The superior catalytic activity can be attributed to two synergistic mechanisms: (1) the cooperative interaction between Fe and Co creating dual active sites for enhanced hydroxyl radical generation, and (2) the electron-donating effect of amino groups facilitating rapid Fe (III)/Fe (II) cycling and efficient H_2_O_2_ activation. Mechanistic studies through intermediate identification elucidated the degradation pathways, confirming the crucial role of -NH_2_ functionalization in accelerating electron transfer and sustaining radical production.

This work provides valuable insights into the rational design of efficient MOF-based Fenton-like catalysts through dual modification strategies. The wide pH adaptability and excellent stability of NH_2_-MOF(Fe, Co) make it particularly promising for practical wastewater treatment applications. Future research should focus on scaling up synthesis procedures, evaluating performance in complex environmental matrices, and developing strategies for catalyst regeneration and recycling. These findings contribute significantly to advancing sustainable solutions for pharmaceutical pollution remediation through advanced oxidation processes.

## Data Availability

The raw data supporting the conclusions of this article will be made available by the authors, without undue reservation.
