# Supplementary material for: Amino-functionalized Fe/Co bimetallic MOFs for accelerated Fe (III)/Fe (II) cycling and efficient degradation of sulfamethoxazole in Fenton-like system
Source: Front Chem. 2025 Mar 25;13:1579108. doi: 10.3389/fchem.2025.1579108 (PMC11986425; doi:10.3389/fchem.2025.1579108)
Supplement: Supplementary file 1 [file DataSheet1.docx]

**Amino-functionalized Fe/Co bimetallic MOFs for accelerated Fe (III)/Fe (II) cycling and efficient degradation of sulfamethoxazole in Fenton-like system**

Xianbing Zhang ^a^, Yuheng Liu ^a^, Jiajia Yuan ^b, *^

^a^ National Engineering Research Center for Inland Waterway Regulation, Chongqing Key Laboratory of Ecological Waterway, Chongqing Jiaotong University

^b^ School of Materials and Environmental Engineering, Shenzhen Polytechnic University, Shenzhen 518055, China

* Corresponding author, Jiajia Yuan, phone: 086-13826596372, Email: 501517876@qq.com

Table.S1. Basic Information of Sulfamethoxazole

| Antibiotics | CAS number | Molecular formula | Relative molecular weight | Molecular Formula | Solubility |
| --- | --- | --- | --- | --- | --- |
| Sulfamethoxazole | 723-46-6 | C_10_H_11_N_3_O_3_S | 253.278 | 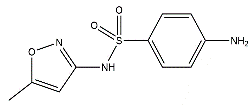 | Freely soluble in dilute hydrochloric acid, sodium hydroxide solution or ammonia, almost insoluble in water |

Table.S2. Raw material compositions for diverse catalysts

| Catalysts | Fe/Co metal ratio | DMF  (mL) | FeCl_3_·6H_2_O  (mmol) | Co(NO_3_)_2_·6H_2_O  (mmol) | H_2_BDC/2-NH_2_-BDC  (mmol) |
| --- | --- | --- | --- | --- | --- |
| MOFs(Fe) | 10:0 | 30 | 2.5 | 0 | 1.25 |
| MOFs(Fe, Co) | 7:3 | 30 | 1.75 | 0.75 | 1.25 |
| NH_2_-MOFs(Fe, Co) | 7:3 | 30 | 1.75 | 0.75 | 1.25 |

Fig.S1 XPS spectra of MOFs(Fe) and MOFs(Fe, Co): (a) C 1s (b) O 1s (c) Fe 2p (d)Co 2p

Text S1 Test methods for the concentration of sulfamethoxazole

Mobile phase A: 0.1% formic acid aqueous solution.

Mobile phase B: methyl alcohol solution.

Mixing ratio: 30%A :70%B

Column temperature: 25℃

Flow rate:0.5mL/min

Detection wavelength: 269 nm

RT: 8 min

Table. S3. Comparative performance of present NH_2_-MOFs(Fe, Co) and other Fenton catalytic systems.

| Target pollutants |  | Catalysts | Reaction conditions | Catalytic performance | References |
| --- | --- | --- | --- | --- | --- |
| Sulfamethoxazole  (SMX) |  | NH_2_-MOFs(Fe, Co) | pH: 5.4; Catalyst: 0.3g/L; [H_2_O_2_]: 1mM; [SMX]: 10mg/L; T: 25℃ | 100% removal in 75 min | This work |
| Sulfamethoxazole  (SMX) |  | MIL-53(Fe) | pH: 4; Catalyst: 2 mmol/L; [H_2_O_2_]: 2mM; [SMX]: 200μg/L; UVA LED irradiation (370nm). T: room temperature; Photo-Fenton | About 96% removal in 120 min | [1] |
| Sulfamethoxazole  (SMX) |  | CUCs-MIL-88B(Fe)/Ti_3_C_2_ | pH: 3; Catalyst: 200mg/L; [H_2_O_2_]: 10mM; [SMX]:30mg/L; T: room temperature Visible light | >96% removal in 120 min | [2] |
| Sulfamethoxazole  (SMX) |  | Fe@MesoC | pH: 4; Catalyst:200mg/L; [H_2_O_2_]: 3mM; [SMX]: 20mg/L; T: 25℃ | 100% removal in 120 min | [3] |
| Sulfamethoxazole  (SMX) |  | Fe_0.75_Cu_0.25_(BDC) | pH: 5.6; Catalyst: 0.5g/L; [H_2_O_2_]: 6mM; [SMX]: 20mg/L; T: 25℃ | 100% removal in 120 min | [4] |

Text S2 Electron paramagnetic resonance technique (EPR) test conditions

Instrument Model: A300-10/12 (Bruker, Germany)

Test Steps: In a 100 ml conical flask 100 ml of hydrogen peroxide solution at a concentration of 1 mM was added, then 30 mg of catalyst was added and timed from this point, waiting for the reaction to take place for 10 min and 20 min.

1)Hydroxyl radicals: take 100 microliters of reaction solution and add 100 microliters of DMPO solution with a concentration of 100 mM, mix well and then use a capillary tube to load the sample and seal the tube, then put it into the sample tube and then put it into the machine to test and collect data.

2) Superoxide radicals: take 50 microliters of reaction solution and add 50 microliters of methanol and 100 microliters of DMPO methanol solution with a concentration of 100 mM, mix well and then use a capillary tube to load the sample to seal the tube, then put it into a sample tube and then put it into a machine to test and collect data.

Text S3 Analytical parameters adopted for SMX intermediates/products.

Instrument conditions

Liquid Chromatography: Agilent Technologies 1290 Infinity II

Mass spectrometry: AB SCIEX TRIPLE TOF 4600

Mobile phase A: 0.1% formic acid aqueous solution; mobile phase B: acetonitrile solution.

| Time[min] | A [%] | B [%] |
| --- | --- | --- |
| 0 | 90 | 10 |
| 2 | 90 | 10 |
| 13 | 5 | 95 |
| 16 | 5 | 95 |
| 16.2 | 90 | 10 |
| 18 | 90 | 10 |

Flow rate: 0.3 mL/min

Injection volume: 50 *uL*

Mass spectrometry scan range: 50-1500 m/z

ESI+ mode

Table. S4. Possible detected intermediates/products of SMX after reaction.

| Number(N) | Possible intermediates | RT (min) | m/z |
| --- | --- | --- | --- |
| 1 | __  Chemical Formula: C_6_H_7_NO_2_ | 8.81 | 129 |
| 2 |   Chemical Formula: C_4_H_6_N_2_O_3_S | 10.22 | 163.04 |
| 3 |   Chemical Formula: C_4_H_10_N_2_O_3_S | 17.3 | 183.08 |
| 4 |   Chemical Formula: C_4_H_6_N_2_O | 8.81 | 99.0553 |
| 5 |   Chemical Formula:C_10_H_11_N_3_O_4_S | 5.54 | 270.0540 |
| 6 |   Chemical Formula: C_10_H_11_N_3_O_4_S | 5.54 | 270.0540 |
| 7 |   Chemical Formula:C_6_H_7_NO_3_S | 8.45 | 173.0878 |
| 8 |  | 17.3 | 256 |
| 9  10 | Chemical Formula: C_9_H_9_N_3_O_4_S    Chemical Formula:C_10_H_9_N_3_O_5_S    Chemical Formula: C_9_H_10_N_3_O_7_S | 5.14  10.22 | 284  304 |

# Reference

1. Ortega-Moreno GA, Ayala-Duran SC, Barbero BP, et al. Photo-Fenton degradation of sulfamethoxazole using MIL-53(Fe) under UVA LED irradiation and natural sunlight. J. Environ. Chem. Eng. 2022;10. https://doi.org/10.1016/j.jece.2022.107678.
2. Ahmad M, Quan X, Chen S, et al. Tuning Lewis acidity of MIL-88B-Fe with mix-valence coordinatively unsaturated iron centers on ultrathin Ti3C2 nanosheets for efficient photo Fenton reaction. Appl. Catal. B Environ. 2020;264. https://doi.org/10.1016/j.apcatb.2019.118534.
3. Tang J, Wang J. Fenton-like degradation of sulfamethoxazole using Fe-based magnetic nanoparticles embedded into mesoporous carbon hybrid as an efficient catalyst. Chem. Eng. J. 2018;351:1085-94. <https://doi.org/10.1016/j.cej.2018.06.169>.
4. Tang J, Wang J. Iron-copper bimetallic metal-organic frameworks for efficient Fenton-like degradation of sulfamethoxazole under mild conditions. Chemosphere. 2020;241. https://doi.org/10.1016/j.chemosphere.2019.125002.
